# Glycosylation pathways in auxin homeostasis

**DOI:** 10.1111/ppl.70170

**Published:** 2025-03-25

**Authors:** Daniela Škyvarová, Federica Brunoni, Asta Žukauskaitė, Aleš Pěnčík

**Affiliations:** ^1^ Department of Chemical Biology, Faculty of Science Palacký University Olomouc Czech Republic; ^2^ Laboratory of Growth Regulators, Faculty of Science Palacký University Olomouc Czech Republic; ^3^ Laboratory of Growth Regulators Institute of Experimental Botany, The Czech Academy of Sciences Olomouc Czech Republic

## Abstract

Auxin glycosylation plays a fundamental role in the regulation of auxin homeostasis, activity, and transport, contributing to the dynamic control of plant growth and development. Glycosylation enhances auxin stability, solubility, and storage capacity, serving as a key mechanism for both temporary inactivation and long‐term storage of auxin molecules. Specific glycosyltransferases are critical for this process, catalyzing glycosylation at either the carboxyl group or the nitrogen atom of the indole ring. The storage roles of glycosylated auxins, such as IAA‐*N*‐Glc, have been shown to be essential during embryogenesis and seed germination, while irreversible conjugation into catabolic products helps to maintain auxin homeostasis in vegetative tissues. This review highlights the diversity, enzymatic specificity, and physiological relevance of auxin glycosylation pathways, including a frequently overlooked *N*‐glycosylation, underscoring its importance in the complex network of auxin metabolism.

## 1 INTRODUCTION

Glycosylation is an important modification found in all eukaryotic organisms (Meech and Mackenzie 1997). This process is catalyzed by glycosyltransferases, which transfer sugar moieties from activated sugar donors (glucose, ribose, galactose, xylose, rhamnose, or glucuronate) to acceptor substrates (Wang et al. 2023). In plants, it governs the activity and abundance of phytohormones, secondary metabolites and toxins (Gharabli et al. 2023). Glycosylation spans across all major phytohormone groups, including auxins, cytokinins, gibberelins, brassinosteroids, abscisic, jasmonic and salicylic acid (Gachon et al. 2005), protecting them from degradation, increasing their polarity and solubility, and facilitating their transport and storage (Gharabli et al. 2023). The loss of this function has also been reported to significantly affect the plant phenotype (Ostrowski and Jakubowska 2014).

Auxins are a group of plant hormones that play a pivotal role in regulating various physiological and developmental processes in plants. These include apical dominance, embryogenesis, suppression of apical root growth, root meristem initiation, tropisms (growth movements), elongation of coleoptile and internode cells, formation of adventitious and lateral roots, and callus cell division in synergy with cytokinins (Sauer et al. 2013). The effects of auxins on plant cells depend highly on their concentration and spatial distribution (Leyser 2006). This dynamic regulation arises from the intricate interplay of various mechanisms, including the metabolic inactivation of auxin molecules by the formation of inactive conjugates, such as glycosides (Ludwig‐Müller 2011).

This review examines auxin glycosylation pathways and their occurrence across plant species, including *N*‐glycosylation, which has received less attention than other conjugation mechanisms. By highlighting the key enzymes, such as glycosyltransferases, and their roles in auxin metabolism, this review clarifies how specific glycosylation pathways regulate auxin activity. Understanding these pathways is essential for uncovering the broader complexity of auxin homeostasis and the dynamic control of plant growth and development.

## 2 METABOLIC PATHWAYS OF AUXINS

Auxins are organic acids characterized by an aromatic ring, indole or phenyl, and a carboxyl side chain. The most prominent and widely studied auxin is indole‐3‐acetic acid (IAA), which was also the very first discovered phytohormone (Paque and Weijers 2016). Other naturally occurring auxins include 4‐chloroindole‐3‐acetic acid (4‐Cl‐IAA) and phenylacetic acid (PAA) (Tivendale et al. 2012; Cook et al. 2016). Indole‐3‐butyric acid (IBA), initially thought to be a synthetic auxin, later on speculated to be a natural auxin and finally discovered to be an endogenous precursor of IAA, serves as a storage and transport form of auxin in plants (Damodaran and Strader 2019). IAA is primarily synthesized from L‐tryptophan (Trp), produced via the shikimate pathway in chloroplasts (Maeda and Dudareva 2012; Tivendale et al. 2014). The first step of this pathway involves the deamination of tryptophan into indole‐3‐pyruvic acid (IPyA), catalyzed by TRYPTOPHAN AMINOTRANSFERASE OF ARABIDOPSIS 1 (TAA1) or TAA1‐RELATED PROTEINS (TARs) (Kasahara 2016). The final step in the biosynthesis of IAA is the oxidation of IPyA, a reaction mediated by monooxygenases belonging to the YUCCA family (Mashiguchi et al. 2011; Stepanova et al. 2011; Won et al. 2011). In addition to the IPyA pathway, plants also utilize alternative interconnected biosynthetic pathways to synthesize IAA through several routes starting from Trp, such as the indole‐3‐acetaldoxime, tryptamine and indole‐3‐acetamide pathways (Tivendale et al. 2014).

Besides de novo biosynthesis, the regulation of free IAA in plants involves oxidative catabolism, conjugation and deconjugation with sugars or amino acids. These processes play a key role in maintaining IAA levels (Ostrowski and Jakubowska 2014), with different species using distinct strategies to achieve it. In monocots, IAA esters are prevalent (Ludwig‐Müller 2011), while IAA amides dominate in dicotyledonous plants (Tam et al. 2000; Bajguz and Piotrowska 2009; Hladík et al. 2023). The synthesis of IAA amides with various amino acids is catalyzed by the GRETCHEN HAGEN 3 (GH3) enzyme family (Staswick et al. 2005; Sugawara et al. 2015). Amide bonds can be cleaved by enzymes such as IAA‐LEUCIN RESISTANT 1 (ILR1), ILR1‐LIKE PROTEINS (ILLs), and IAA‐ALANINE RESISTANT (IAR3) (LeClere et al. 2002). Besides IAA, GH3 enzymes also conjugate other auxin phenylacetic acid (PAA) as well as other phytohormones such as jasmonic acid and its precursor cis‐(+)‐12‐oxo‐phytodienoic acid (cis‐OPDA) (Staswick et al. 2005; Hladík et al. 2024; Široká et al. 2025).

Auxin oxidative degradation is catalyzed by the enzyme DIOXYGENASE FOR AUXIN OXIDATION (DAO) (Zhao et al. 2013; Porco et al. 2016; Zhang et al. 2016b). Oxidation of IAA at the C2 position produces the irreversible metabolite 2‐oxindole‐3‐acetic acid (oxIAA) (Östin et al. 1998; Pěnčík et al. 2013). The activity of DAO is critical for processes like pollen fertility and seed germination in rice (Zhao et al. 2013). oxIAA can be further conjugated with glucose (Tanaka et al. 2014; Brunoni et al. 2019), forming oxIAA‐Glc, an abundant metabolite in Arabidopsis seedlings (Kai et al. 2007a; Hladík et al. 2023) or with amino acids, forming oxIAA‐amino acids (oxIAA‐AAs) (Brunoni et al. 2023). Amide conjugates, such as IAA‐Asp and IAA‐Glu, are also prone to oxidation, resulting in inactive oxIAA‐AAs (Kai et al. 2007a; Hayashi et al. 2021; Müller et al. 2021). Another oxidized metabolite, 3‐hydroxy‐oxIAA (dioxIAA), is also an inactive and irreversible auxin form, though its formation mechanism remains unknown (Isobe and Miyagawa 2022). In flowering plants, oxidative inactivation of IAA and IAA‐AAs plays a predominant role. In contrast, gymnosperms primarily rely on GH3‐mediated conjugation of IAA and oxIAA with amino acids for auxin inactivation. This indicates that the relative contribution of conjugative and oxidative pathways to IAA homeostasis is species‐dependent (Brunoni et al. 2020, 2023).

IAA can also be inactivated through methylation, where IAA CARBOXYL METHYLTRANSFERASE 1 (IAMT1) converts IAA to its inactive methyl ester form MeIAA, which is important for processes like leaf development and gravitropism (Qin et al. 2005; Korasick et al. 2013; Abbas et al. 2018). METHYLESTERASE 17 (MES17) can then conversely release active IAA from the methyl ester, allowing it to participate in auxin signaling again (Yang et al. 2008).

Finally, IAA can be glycosylated at either the nitrogen atom of the indole ring or the oxygen atom of the carboxyl group. Through conjugation with sugar, IAA gains greater stability and water solubility, which facilitates its transport and storage (Jones and Vogt 2001). It is believed that the *N*‐glucoside of IAA (IAA‐*N*‐Glc) is more stable than the *O*‐glucosyl ester (IAA‐Glc) (Yin et al. 2021). For IAA to be reactivated from its glucose conjugate, synthesis of hydrolytic enzymes is necessary during seed development and maturation (Kai et al. 2007b). However, IAA glycoside hydrolases have not yet been fully identified. The only known hydrolase thus far is *THOUSAND‐GRAIN WEIGHT 6* (TGW6) from rice (Ishimaru et al. 2013; Akabane et al. 2024).

## 3 AUXIN SUGAR CONJUGATES

### 3.1 Auxin glycosyl esters

IAA glycosyl esters primarily serve as storage compounds. They are most commonly formed by esterification of the carboxyl group of IAA with glucose, catalyzed by specific uridine diphosphate (UDP)‐glucosyl‐transferases (UGTs) (Szerszen et al. 1994), resulting in glucosyl esters. Similarly, oxIAA‐Glc is synthesized via the glucosylation of oxIAA, not through the oxidation of IAA‐Glc (Tanaka et al. 2014; Porco et al. 2016; Hayashi et al. 2021). Active IAA can be released from the ester metabolite through the action of hydrolases (Ishimaru et al. 2013; Aoi et al. 2020). IAA esters are the main conjugated form of auxin in the seeds of many economically important crops, including maize, rice, and wheat (Bandurski and Schulze 1977), where they serve as a primary source of active IAA during germination. The endogenous presence of IAA‐Glc and oxIAA‐Glc has been repeatedly confirmed in *Arabidopsis thaliana*, with oxIAA‐Glc being the major conjugate among all low‐molecular‐weight IAA metabolites identified so far (Porco et al. 2016; Pěnčík et al. 2018; Široká et al. 2022; Hladík et al. 2023). In Arabidopsis seedlings, IAA‐Glc predominantly accumulates in root tissues (Ljung et al. 2001; Mateo‐Bonmatí et al. 2021). In addition to Arabidopsis, IAA‐Glc has been detected in spruce, the vegetative parts of pea, but not in maize or wheat (Široká et al. 2022; Hladík et al. 2023). Moreover, IAA‐Glc, but not oxIAA‐Glc, is also the most abundant IAA metabolite in conifer seedlings (Brunoni et al. 2020), while oxIAA‐Glc, but not IAA‐Glc, has been reported in *Brassica rapa* and *Physcomitrium patens* (Široká et al. 2022).

Besides glucosyl ester conjugates, several other sugar conjugates have also been identified. In maize, the ester conjugate IAA‐*myo*‐inositol is an important source of IAA during seed germination (Hall 1980; LeClere et al. 2002), regulates auxin levels during seedling development and plays a role in drought and cold stress responses (Ciarkowska et al. 2023). Furthermore, plants have been found to contain IAA‐glycans and IAA‐glycoproteins, although their function remains unclear (Korasick et al. 2013).

### 3.2 Auxin *N*‐glucosides

IAA possesses both a carboxyl group and an indole ring, readily providing multiple sites for glucosylation. While the carboxyl group is a common target, the nitrogen atom of the indole ring can also be glucosylated. Although the corresponding products IAA‐Glc and IAA‐*N*‐Glc share the same molecular weight, they can be distinguished using mass spectrometry based on differences in retention time and the resulting molecular fragments. This approach, combined with the use of alkaline hydrolysis, effectively cleaving glucose from the glucosyl ester but not from the *N*‐glucoside, aided the discovery of IAA‐*N*‐Glc and its amide conjugate IAA‐Asp‐*N*‐Glc in *Pinus sylvestris* (Ljung et al. 2001).

IAA‐*N*‐Glc was further detected in maize (Kai et al. 2007b), fungus (*Cortinarius brunneus*) (Teichert et al. 2008), red and white currants (Schwarz and Hofmann 2007; Godjevac et al. 2011), and seeds of *Ginkgo biloba* (Yin et al. 2021) and Chinese horse chestnut (*Aesculus chinensis var. chekiangensis*) (Zhang et al. 2019). In rice, its conjugated forms with aspartic and glutamic acids, IAA‐Asp‐*N*‐Glc and IAA‐Glu‐*N*‐Glc, respectively, were observed in vegetative tissues, while free IAA‐*N*‐Glc was detected only in seeds. During the vegetative growth of rice seedlings, the levels of IAA‐*N*‐Glc conjugates increased, while the levels of IAA‐Asp and IAA‐Glu conjugates decreased. Throughout rice development, the levels of IAA‐AA‐*N*‐Glc were significantly higher than those of IAA‐AA. In both cases, conjugates with Asp predominated over those with Glu. Interestingly, the levels of both IAA‐Asp‐*N*‐Glc and IAA‐Glu‐*N*‐Glc were significantly higher in roots than in aerial parts (Kai et al. 2007b). The pronounced accumulation of these IAA‐AA‐*N*‐Glc conjugates, particularly in roots, suggests that they may serve as a long‐term storage or detoxification form of auxin, potentially playing a role in regulating spatial auxin gradients through inter‐tissue transport or local synthesis. Both IAA‐Asp‐*N*‐Glc and IAA‐Glu‐*N*‐Glc were also detected in the seeds of *Ginkgo biloba* and reported for their anti‐inflammatory activity (Cheng et al. 2020).

The storage function of IAA‐*N*‐Glc and its role in the early stages of embryo development is inferred from its presence in seeds. It is likely that IAA‐*N*‐Glc supplements or replaces the storage role of IAA esters during germination. During early embryonic development, cells divide and then elongate, forming the basis of seedling initials (Sandberg et al. 1987). This process is correlated with IAA metabolism, which mediates cell elongation. An experiment with germinating *Pinus sylvestris* seeds revealed dynamic changes in IAA and its metabolite composition during various stages of seedling development. Free IAA increased in the first 48 hours after the start of swelling, correlating with a decrease in storage conjugates in the seed, suggesting hydrolytic release of active IAA, hence eliminating the need for de novo IAA synthesis in the early stage. After 48 hours, the level of free IAA began to decrease, initiating root elongation and hypocotyl formation, while de novo IAA biosynthesis from tryptophan began by day four, triggering catabolism. Between days four and six, all storage conjugates were depleted, and the first products of IAA metabolism, such as amide conjugate IAA‐Asp, emerged. However, this was rapidly glucosylated into IAA‐Asp‐*N*‐Glc, which no longer serves as a storage form but as an irreversible catabolite (Ljung et al. 2001). In combination with isotopic labeling experiments, this suggests that *N‐*glucosides may have a dual role: acting as catabolites during vegetative growth and potentially functioning as storage forms of auxin in seeds.

In *Ginkgo biloba*, the formation of IAA‐AA‐*N*‐Glc can occur in two ways: either by forming the amide conjugate followed by glucosylation, involving enzymes GbGH3.5 and GbNGT1, or by glucosylation followed by the addition of the amino acid. The formation of IAA‐AA‐*N*‐Glc through glucosylation from IAA‐AA was confirmed by the addition of deuterium‐labeled IAA‐AA in rice (Kai et al. 2007b). In a test with *Ginkgo biloba* mutants with a silenced GbNGT1 gene, it was shown that the *N*‐glucosyltransferase GbNGT1 enables glucosylation of IAA‐AA conjugates at the nitrogen atom of the indole ring (Yin et al. 2021). Thus, *N*‐glucosylation of IAA‐AA conjugates is an inactivation process.

Additionally, oxIAA‐*N*‐Glc and dioxIAA‐*N*‐Glc have been identified in the seeds of *Ziziphus jujuba* var. *spinosa* (Li et al. 2014, 2021; Zhang et al. 2016a; Chen et al. 2020c); however, no studies thus far have elucidated their role in auxin homeostasis or identified the enzymes involved in their biosynthesis.

### 3.3 Other auxin *O*‐glucosides

IAA *O*‐glucosides, in which glucose is attached to the indole ring of dioxIAA or other hydroxylated IAA metabolites through *O*‐glucosidic linkage, represent probably the most underexplored class of auxin conjugates. Albeit sometimes only tentatively, such *O*‐glucosylated forms have been detected in plants.

Through feeding experiments, dioxIAA‐Asp‐3‐*O*‐Glc has been identified in broad bean (*Vicia faba* L.) and *Dalbergia dolichopetala* seedlings (Tsurumi and Wada 1986; Östin et al. 1992) and oxIAA‐7‐*O*‐Glc was identified in *Zea mays* seedlings (Nonhebel and Bandurski 1984; Nonhebel et al. 1985). dioxIAA‐5‐*O*‐Glc and dioxIAA‐7‐*O*‐Glc were isolated from corn (*Zea mays* L.) kernels (Tateishi et al. 1987, 1988, 1989), while both dioxIAA‐5‐*O*‐Glc and dioxIAA‐5‐*O*‐Glc‐Glc were isolated from rice bran (Tateishi and Yamashita 1998). More recently, dioxIAA‐3‐*O*‐Glc has been isolated from the fruits of mulberry (*Morus alba* L.) (Yu et al. 2018), sour cherries (*Prunus cerasus* L.) (Piccolella et al. 2008) and hazelnut kernels (*Corylus avellana* L.) (Singldinger et al. 2018; Shataer et al. 2021) and oxIAA‐5‐*O*‐Glc has been tentatively identified in jackfruit (*Artocarpus heterophyllus* Lam.) peel (Zhang et al. 2017).

Though acknowledged, up to now, both auxin hydroxylation and subsequent *O*‐glucosylation, through which glucose is conjugated to the hydroxyl group, remain largely unexplored (Kai et al. 2007a; Zhang and Peer 2017).

## 4 AUXIN GLUCOSYLTRANSFERASES

UGTs are a family of enzymes that catalyze the transfer of glucose from UDP‐glucose, a high‐energy sugar donor, to acceptor molecules such as hormones and secondary metabolites (Gachon et al. 2005). This process enhances the target molecules' solubility, stability, and transportability. In plants, UGTs play a critical role in regulating phytohormone levels, including auxins, by converting them into glucosylated conjugates through the formation of *O*‐glucosidic bonds (Gharabli et al. 2023). UGTs contain a His19/20 residue responsible for removing a proton from the carboxyl group of the phytohormone, forming an oxyanion that enables the nucleophilic attack of the C1 carbon of glucose on the hydroxyl group (Wang 2009). These enzymes exhibit remarkable substrate specificity (Osmani et al. 2009) and are essential for maintaining hormonal homeostasis, responding to environmental stimuli, and controlling developmental processes (Gharabli et al. 2023). In contrast, at least in the field of auxin research, the same level of knowledge cannot yet be claimed for *N*‐glucosyltransferase (NGT)‐mediated *N*‐Glc modifications.

The first identified gene encoding an auxin‐conjugating UGT was *ZmIAAGLU* (*IAGLU*) in maize, and the presence of homologous genes was also suggested in *Arabidopsis thaliana*, cauliflower, duckweed, tomato, rice, soybean, and tobacco (Szerszen et al. 1994). IAGLU in maize influences leaf morphology and flower development. To further investigate *UGT* genes, model plants such as *Arabidopsis thaliana* and rice were used. A total of 115 *UGT*‐encoding genes were identified in Arabidopsis and 200 in rice (Yu et al. 2017), of which 12 in rice were specific to auxins. One of them, *OsIAGT1*, encodes a UGT with the highest activity at pH 8 and greater substrate affinity for IAA and IBA. The expression of this gene is induced by auxin and is localized in mature leaves and stems, where high concentrations of active IAA are no longer required. In young leaves, *OsIAGT1* is expressed to a much lesser extent. Overproduction of OsIAGT1 resulted in reduced rice growth (Liu et al. 2019). Overexpression of *UGT* genes also increased the levels of active IAA, suggesting the existence of hydrolases that cleave IAA‐Glc (Jackson et al. 2002).

**FIGURE 1:**
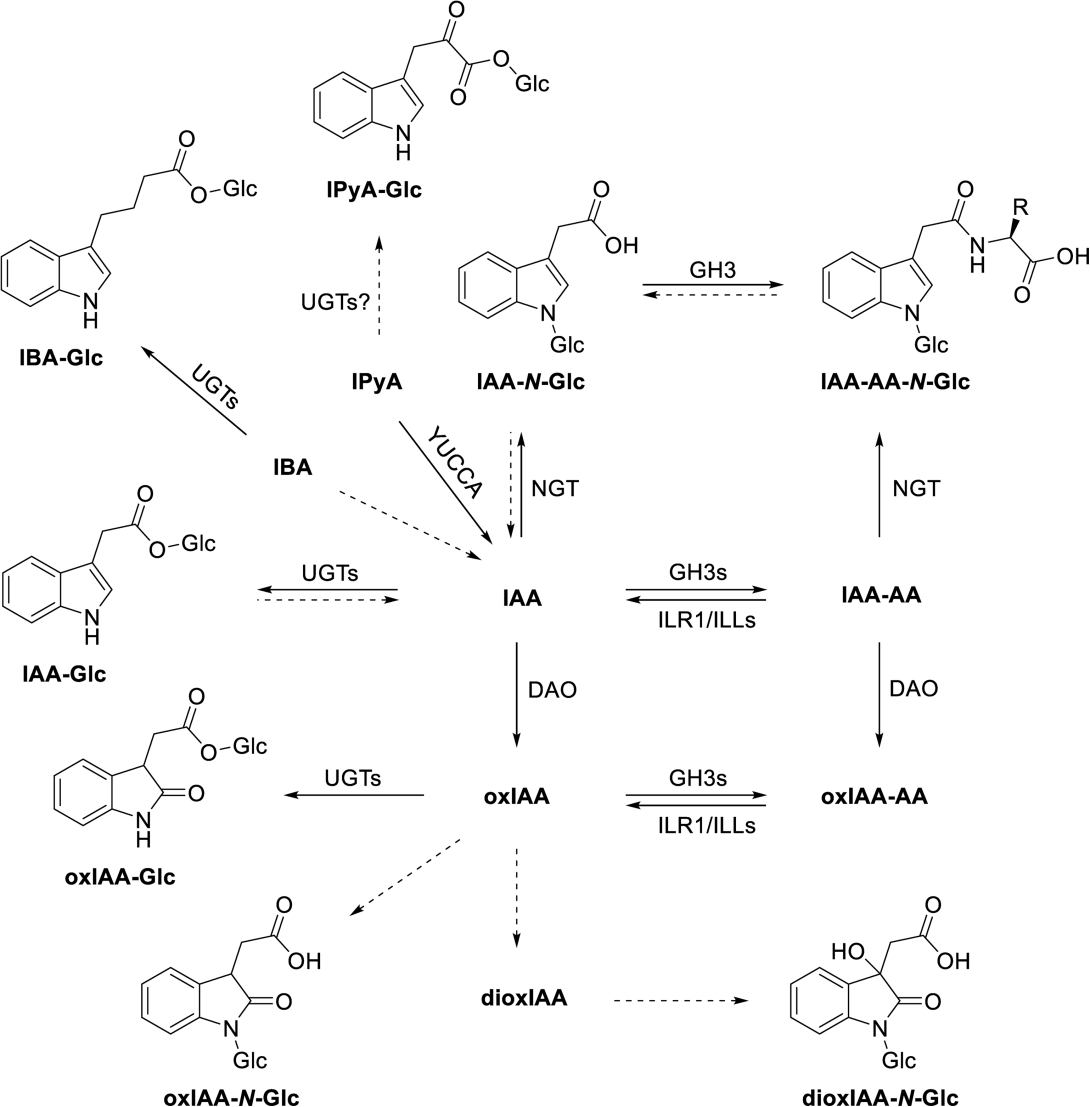
Glycosylation pathways of IAA, its metabolites, and precursors. Dashed lines represent reactions catalyzed by unknown enzymes. Based on the following publications: Jackson et al. 2001; Ljung et al. 2001; Jin et al. 2013; Brunoni et al. 2019, 2023; Chen et al. 2020a; Mateo‐Bonmatí et al. 2021; Hayashi et al. 2021; Yin et al. 2021; Harada et al. 2024.

There is a wide spectrum of UGTs with highly diverse substrate specificity (Figure 1, Table 1). UGT84B1, UGT84B2, UGT75B1, UGT75B2, and UGT74D1 were found to glucosylate IAA and other natural and synthetic auxins in *Arabidopsis thaliana* (Jackson et al. 2001; Jin et al. 2013; Brunoni et al. 2019; Hladík et al. 2024). UGT84B1 can catalyse glucosylation of oxIAA (Brunoni et al. 2019; Mateo‐Bonmatí et al. 2021). Moreover, UGT84B1 has been shown to glucosylate PAA in vitro (Aoi et al. 2020). UGT74D1 catalyzes the glucosylation of IAA and oxIAA and has higher expression in vegetative tissues (Jin et al. 2013; Tanaka et al. 2014). The most active enzymes in several plant species are UGT74D1 and UGT84B1, with UGT84B1 being the most active in *Arabidopsis thaliana* (Jackson et al. 2001, 2002; Jin et al. 2013; Mateo‐Bonmatí et al. 2021). Furthermore, four paralogs of the UGT76E3456 subfamily were found to play a role in hypocotyl growth in darkness by modulating IAA levels through glucosylation of the catabolite oxIAA, supporting differential and developmental stage‐specific contributions of the UGT84B1, UGT74D1, and UGT79E3456 glucosyltransferases to IAA metabolism by mediating IAA and oxIAA glucosylation (Mateo‐Bonmatí et al. 2021). UGT76F2, earlier annotated as UGT76F1, was initially believed to regulate IAA biosynthesis in the hypocotyl by inactivating its precursor IPyA. However, a recent study utilizing a newly generated *ugt76f2* mutant has demonstrated that UGT76F2 is not involved in IPyA glucosylation (Chen et al. 2020b, 2020a; Harada et al. 2024). UGT74E2 catalyzes the glucosylation of IBA (Jin et al. 2013). Disruptions in the functionality of these enzymes cause damage to shoot architecture and impair stress responses (Liu et al. 2019).

**TABLE 1:** Overview of auxin glycosyltransferases.

	Glycosyltransferase	Substrate						Reference
		IAA	IAA‐AA	oxIAA	IBA	PAA	IPyA	
NGTs	GbNGT1/UGT717A2	×	×					(Yin et al. 2021)
UGTs	OsIAGT1/OsIAGLU	×			×			(Liu et al. 2019)
	AtUGT74D1	×		×	×	×		(Jin et al. 2013; Tanaka et al. 2014; Hladík et al. 2024)
	AtUGT74E2				×			(Tognetti et al. 2010)
	AtUGT75B1	×			×			(Jackson et al. 2001)
	AtUGT75B2	×			×			(Jackson et al. 2001)
	AtUGT76E3456			×				(Mateo‐Bonmatí et al. 2021)
	AtUGT84B1	×		×	×	×		(Jackson et al. 2001; Brunoni et al. 2019; Hladík et al. 2024)
	AtUGT84B2	×			×			(Jackson et al. 2001)
	AtUGT76F2/UGT76F1						?	(Chen et al. 2020b, 2020a; Harada et al. 2024)
	ZmIAGLU/IAGLU	×						(Szerszen et al. 1994)

So far, the only exclusive NGT identified is GbNGT1/UGT717A2 in *Ginkgo biloba*. Overexpression of GbNGT1 in *Arabidopsis thaliana* induced over‐formation of IAA‐*N*‐Glc and promoted root growth, likely due to the mediation of IAA distribution to the root cap. GbNGT1 was able to specifically *N*‐glucosylate IAA, IAA‐Asp, IAA‐Glu, IAA‐Leu and IAA‐Gly to form corresponding IAA‐*N*‐Glc and IAA‐AA‐*N*‐Glc conjugates, while even minimally modified IAA derivatives or structurally similar IBA or indole‐3‐propionic acid (IPA) were not accepted as substrates, highlighting its strong specificity (Yin et al. 2021). A similar function in eukaryotes is carried out by oligosaccharyltransferase (OST), which is not related to NGT. OST catalyzes the formation of a bond between the amide of aspartic acid on a protein side chain and the C1 carbon of the sugar's reducing end (Naegeli et al. 2014).

## 5 CONCLUSIONS AND PERSPECTIVES

Understanding the complex interplay of auxin glycosylation pathways within the broader regulatory network of auxin metabolism represents a significant challenge in plant biology. While substantial progress has been made in characterizing the GH3‐ILR1‐DAO regulatory framework for IAA inactivation, the role of glycosylation as a complementary or alternative pathway remains underexplored. Auxin glycosylation is essential for seed germination and plays a complex role during vegetative growth. Herein, we highlight key unresolved questions and emerging directions that aim to advance our understanding of auxin homeostasis across diverse plant lineages:

Q1) The GH3‐ILR1‐DAO regulatory framework has been proposed as the main inactivation pathway of IAA in flowering plants (Hayashi et al. 2021). However, IAA‐Glc and oxIAA‐Glc are among the most abundant IAA derivatives in Arabidopsis (Östin et al. 1998; Pěnčík et al. 2013; Porco et al. 2016), which raises a question: how does IAA glycosylation complement the GH3‐ILR1‐DAO inactivation pathway? Moreover, while IAA glycosylation and hydrolysis of IAA glucosides are pivotal during seed germination (Ljung et al. 2001), IAA glucosides are also detected at high levels during vegetative growth (Porco et al. 2016; Mateo‐Bonmatí et al. 2021). This raises further questions about how IAA glycosylation contributes to maintaining IAA homeostasis during later developmental stages.

Q2) How do glycosylation pathways contribute to maintaining auxin homeostasis in nonflowering plants, such as gymnosperms? Considering that conjugative pathways are the preferred strategy adopted by conifers for fine‐tuning basal IAA concentrations (Brunoni et al. 2020, 2023), it might be that IAA glycosylation routes are the most relevant means of IAA inactivation in this plant lineage.

Q3) Algae like charophytes and primitive land plants like moss and liverworts rely on simpler strategies for managing excess IAA, balancing biosynthesis and degradation (Ester Sztein et al. 1999; Cooke et al. 2002; Záveská Drábková et al. 2015). The evolution of more sophisticated mechanisms, including IAA conjugation and conjugate hydrolysis, raises the question of when and how IAA glycosylation first emerged. Moreover, considering the critical role of IAA glycosylation in homeostasis during seed germination and its dependence on the UGT multigene family, which is present across organisms from bacteria to humans (Mackenzie et al. 1997), the question arises on how IAA glycosylation evolved.

Q4) How are IAA‐*N*‐Glc and IAA‐AA‐*N*‐Glc formed? Which enzymes are responsible for their synthesis and hydrolysis? What is the overall contribution of these pathways to the regulation of IAA homeostasis?

Q5) Since sugar conjugation enhances auxin stability, water solubility and has been proposed as a biological tagging mechanism that regulates metabolite activity and compartmentalisation, how does IAA glycosylation contribute to regulating auxin intracellular homeostasis? High‐resolution profiling of IAA metabolites is expected to significantly enhance our understanding of how these metabolites influence auxin homeostasis. Achieving this requires ready availability or the synthesis of analytical standards, including those of well‐characterized auxin metabolites and those thus far tentatively identified or even unknown. These reference compounds will help to support the identification of these metabolites, confirm their biosynthetic pathways, and enable precise quantification of their abundance in *Arabidopsis* and other phylogenetically distant species.

Q6) How does auxin glycosylation link auxin with primary metabolism? IAA affects primary metabolism and vice versa, but the underlying mechanisms are still poorly understood (Tivendale and Millar 2022). Considering that the chloroplast metabolism alters IAA synthesis through the synthesis of Trp, the main IAA precursor, and that the synthesis of sugars also occurs in the chloroplast, the regulation of IAA levels via glycosylation might represent an additional connection between chloroplast function and IAA homeostasis.

The investigation of these questions is inherently complex and multifaceted, as redundant pathways often operate in parallel to regulate auxin homeostasis. Such investigation requires a multidisciplinary approach that combines expertise in organic and analytical chemistry, genetics, and other fields. Efforts in this direction are expected to deepen our understanding of auxin homeostasis by shedding light on underexplored pathways and further illuminating their pivotal role in the dynamic control of plant growth and development.

## AUTHOR CONTRIBUTIONS

AŽ, FB, AP: Conceptualization. AŽ: Visualization. DŠ: Writing ‐ Original Draft. FB, AŽ, AP: Writing ‐ Review & Editing. All authors read and approved the final manuscript.

## FUNDING INFORMATION

The work was supported by the project TowArds Next GENeration Crops (TANGENC), reg. no. CZ.02.01.01/00/22_008/0004581, of the ERDF Programme Johannes Amos Comenius and by the Internal Grant Agency of Palacký University Olomouc (IGA_PrF_2025_013).

## Data Availability

N/A
